# Values, Environmental Beliefs, and Connection With Nature as Predictive Factors of the Pro-environmental Vote in Spain

**DOI:** 10.3389/fpsyg.2020.01043

**Published:** 2020-06-03

**Authors:** M. Carmen Aguilar-Luzón, Beatriz Carmona, Antonia Calvo-Salguero, Pedro A. Castillo Valdivieso

**Affiliations:** ^1^Department of Social Psychology, University of Granada, Granada, Spain; ^2^Department of Architecture and Computer Technology, University of Granada, Granada, Spain

**Keywords:** pro-environmental parties, voting behavior, values, ecocentrism, anthropocentrism

## Abstract

This research analyzes the predictive capacity of psychosocial variables that can influence the decision to vote for political parties that include pro-environmental measures in their program. To this end, a study was carried out with a sample of 414 people of legal age who could exercise their right to vote (mean age = 26.92, *SD* = 10.53). The participants were divided into two groups: (1) Pro-environmental voters, those who during the last elections in Spain based their voting decision on whether the political party included pro-environment measures in its electoral program (*n* = 190), and (2) Non-pro-environmental voters, those other people who voted for a political party without considering whether pro-environment measures were included in its electoral program (even if such environmental protection measures had been included) (*n* = 224). The results indicate that, in comparison with their counterparts who do not vote for pro-environmental parties, those who voted for political parties during the last elections by considering the inclusion of pro-environment measures in their electoral program showed the highest scores on the biospheric and socio-altruistic values of ecocentrism, anthropocentrism, connectivity with nature and environmental concern, and scored lower on self-centered values. With the exception of connectivity with nature, biospheric values and beliefs were good predictors of pro-environmental voting behavior.

## Introduction

It is accepted that we currently live in a time in which there are fears for the environment due to the increasing deterioration of the planet. According to 2019 Eurobarometer data, the economy and growth was the biggest issue for voters in 16 Member States, while climate change and the environment was the main issue in eight countries. A simple example of this can be seen in the Eurobarometer conducted by the European Parliament, which states that two thirds of Europeans want the European Union to show a stronger commitment to environmental protection, and in Spain this proportion rises to 78% of the respondents. Since the World Conference on the Human Environment (1972, Stockholm Conference) took place, the environment has been considered the axis of international policies. However, the reality is that, even today, governments and states have not been able to reach the level of commitment required by the so-called pro-environmental economy in order to achieve sustainable development. Thus — and as stated in the recent report presented by the Intergovernmental Panel on Climate Change (IPCC) — far-reaching and unprecedented changes would be needed in all aspects of society in order to ensure that the impact of global warming on our planet is kept to a minimum (IPCC, 2018). As already indicated by Monge (2013) “the global environmental crisis implies a change in the patterns of production and consumption, a new economy for a new model of development, a pro-environmental economy that is capable of ensuring decent living and working conditions, while reducing risks and environmental degradation” (pp. 53).

In this scenario, therefore, there is a need for participation and involvement, not only on the part of the governments of the nations, but of civil society, if we are to curb the current global crisis derived from the models of production and overconsumption that have been generated by capitalists. Given this context, psychology has been dedicated to the study of both individual variables (e.g., values, beliefs, and attitudes), and social variables (e.g., social and demographic factors) involved in the implementation of actions that favor the environment, or so-called pro-environmental behaviors (Herrera-Mendoza et al., 2016). However, whilst various studies report that knowledge regarding the destruction of the planet and the sensitivity toward the improvement and protection of the environment have increased among the general population, this does not appear to have translated into specific behaviors on the part of society (Álvarez and Vega, 2009). In fact, a recent study highlights the need to analyze the mechanisms that lead people to acquire so-called frugal behavior, that is, those behaviors characterized by a reduction in consumption that implies the austere use (or non-waste) of services and material goods, taking into account the positive and rewarding consequences that this implies in the face of materialistic values (Hernández et al., 2018). Thus, if society as a whole were to exhibit behaviors as a result of a rational decision-making process, this could help to maintain the social practices of saving and preserving resources. These behaviors include those individual actions — direct or indirect — that seek to preserve, conserve or, at least limit the level of harm caused to the environment, such as, for example, the purchase of organic products, environmental activism, reuse or recycling, and the saving of water and energy (López et al., 2015) to name but a few.

Another behavior that could be considered to fall under the umbrella of environmental behaviors is the green vote. Although voting behavior is considered by some authors to be political behavior (Sartori, 1992; Salamanca, 2012), we could also consider it an environmental behavior, in that it is a matter of casting a vote in favor of parties that plan to adopt economic measures — both social and political — in favor of the environment, that is, the so-called *green parties*. From our point of view, this action should therefore be regarded as an indirect environmental action that reflects the attitudes and desires of the individual to maintain a long-term social-environmental framework that allows citizens to behave in ways that are respectful of the physical and/or social environment.

From our point of view, this action should therefore be regarded as an indirect environmental action that reflects the attitudes and desires of the individual to maintain a long-term social-environmental framework that allows citizens to behave in ways that are respectful of the physical and/or social environment.

However, the majority of research aimed at studying voting behavior has been developed in the context of analyzing the relationship between this behavior and variables linked to economy-employment or safety-violence, largely ignoring the analysis of dimensions related to ecological issues. In this regard, after carrying out a bibliographic review in specialized journals, we were unable to find published empirical works that have explicitly aimed to identify the characteristic psychosocial profile of a pro-environmental party voter and/or analyze the predictive power of the variables involved in pro-environmental voting behavior. We can only highlight the work of Cowie et al. (2015) who identified different subgroups of pro-environmental voters following the general election of New Zealand in 2011, in which the Pro-environmental Party experienced unprecedented support. Using data from the New Zealand Attitudes and Values Study (2012), they found, in different subpopulations of pro-environmental voters, disparities regarding essential values such as those linked to social justice and the rights of the Maori; however, all of these converged on environmental values.

It is possible that the absence of work on this topic could be due to the difficulties involved in obtaining adequate samples of pro-environmental voters, since in a broader cultural context, voters who sympathize with pro-environmental parties, sustainable platforms, or ecological proposals are generally considered to be minorities (Carreón et al., 2014).

The present work, therefore, constitutes an approach to the analysis of voting behavior when voters take into account the environmental issues included in the electoral program of a political party that aspires to govern a state. We believe that this study could have important implications for the decision-making of political parties when designing and preparing their electoral campaigns, whilst allowing for a deeper understanding of the variables involved in the decision to vote for a pro-environmental party.

To this end, we have considered the role played by those variables that are most frequently identified in the literature as being closely linked to environmental behaviors, including ecological voting behavior. Among these variables, both cognitive and emotional nature have been considered, since both types of variables have been linked to political voting behavior. From the rational theories that analyze voting behavior, the voter is considered to be a rational being and, therefore, his/her political behavior is the result of the analysis or reasoning about the advantages, disadvantages, benefits and risks that are involved in making a particular decision (Valdez and Huerta, 2011). From the perspective that considers the influence of emotional factors on voting behavior, political attitudes and actions, such as voting for a political party, are determined by the affect (Becerra, 2016). Therefore, the variables analyzed in this study (values, environmental system beliefs, including ecocentrism, anthropocentrism, and connectivity with nature) appear to be critical for explaining the behavior of the pro-environmental voter, not only because of their link to environmental behavior, but also because of their cognitive and emotional nature. In the following sections we describe the main results that have been found in the previous literature with regard to these variables.

### Values

Personal values play an important role in the cognitive analysis of the costs and benefits associated with an action (Payne et al., 1992), since they serve to guide behavior and influence the attitudes generated (Rokeach, 1968). Thus, the value orientations held by an individual will have an influence on beliefs, and therefore on attitudes and behaviors, since they act as a filter that modulates the information that the person will evaluate, so that if the available information about the situation, object, or the behavior itself is consistent with the individual’s values, that person will develop more positive beliefs toward that situation, object, or action (Stern and Dietz, 1994; Stern et al., 1995a, b, 1998). For instance, Aguilar-Luzón et al. (2006, 2008) studied how personal values are related to pro-environmental behavior. The results reported by these authors showed that biospheric values (those related to showing concern for non-human species and for the biosphere as a whole), and to a lesser extent socio-altruistic values (related to the concerns shown for other people) were good predictors of the tendency for people to carry out pro-environmental actions, while values of a selfish nature (those related to self-concern) presented a significant and negative relationship with such behavioral intentions. A more recent study indicates that biospheric values are positively and significantly associated with both the intention to carry out a determined pro-environmental behavior (glass recycling), and the behavior itself. Egocentric values, on the other hand, show a significant and negative relationship with said behaviors and intentions (Carmona-Moya et al., 2017).

Values have also been studied in the field of politics and have been shown to underlie political attitudes whilst implicitly influencing people’s political orientations and voting preferences (Caprara et al., 2012). For example, Solano et al. (2015) carried out a study on political behavior in order to determine if there were differences in identity and values in relation to the vote cast in the 2011 Peruvian elections. In terms of the differences in values, it was found that the participants who had voted for the left-wing candidate (Ollanta Moisés Humala Tasso, Peruvian Nationalist Party), showed a greater orientation toward the dimension of socio-altruistic values (including values such as helping, sharing, and being altruistic), while those participants who voted for the candidate proposing a neoliberal economic model in Peru (Keiko Fujimori, Popular Force Party) presented significant differences from the other voters, showing a stronger orientation toward the dimension of self-centered values (among which the authors included values such as discipline, patriotism, perseverance, and success). Therefore, it appears that values are an important variable when explaining an individual’s voting intentions (Barnea and Schwartz, 1998; Caprara et al., 2006).

### Environmental Beliefs

Another component frequently studied in the analysis of environmental behavior is the belief system. One of the most widely used measures for the study of environmental beliefs is the scale developed under the new ecological paradigm (NEP) by Dunlap et al. (2000). These general beliefs toward environmentalism — as shown in most of the studies conducted since the mid-1970s until now — are related to the way in which people are predisposed to act for or against the environment. In particular, a review of the literature reveals empirical evidence showing that those people who have a greater predisposition to act in favor of the environment are those who adhere more strongly to ecocentric beliefs (e.g., issues concerned with biospheric aspects that emphasize the intrinsic properties of nature) and less to anthropocentric-type beliefs (e.g., beliefs about the psychological and physical benefits that nature produces for the human being; Amérigo et al., 2005, 2013; Vozmediano and San Juan, 2005; Calixto and Hernández, 2012; López et al., 2015).

Regarding the role of beliefs in political behavior, there are data suggesting that the beliefs that society has about itself, about nature, and the relationship that unites them, all have an impact on a person’s voting intentions. In this regard, Carreón et al. (2014) under the assumptions of the new ecological paradigm (NEP) and the social theory of post-materialism, carried out a study in which they analyzed how psychological and environmental factors affect the political paradigm. The results obtained showed that beliefs about the environment and the actions of the government were determinants of support for one candidate or another during the elections. In particular, those who believed in the existence of environmental deterioration showed greater support for parties with pro-environmental proposals and they had the expectation that government action would change this problem. In this line, in the present investigation we have also considered the role played by environmental beliefs (ecocentrism vs. anthropocentrism) when deciding to vote in favor of pro-environmental parties. Anthropocentrism is associated with ideas of progress and productivity without limiting human growth and considers that humans are superior to the rest of nature. This anthropocentric dimension of environmental beliefs implies a vision of the natural environment based on the material benefits that this can bring to humans. In contrast, the polar opposite of anthropocentrism is ecocentricism, that is, beliefs based on a vision of nature itself that human beings are just one of its many elements (Thompson and Barton, 1994; Aguilar-Luzón et al., 2014). In this sense, some studies have suggested that stronger ecocentric beliefs should imply a higher level of commitment to caring for nature (González and Amérigo, 1999; Pato et al., 2005; Vozmediano and San Juan, 2005).

### Connectivity With Nature

Recent research highlights the need to consider the role played by certain variables that are linked to the emotional aspects of environmental behavior (Amérigo et al., 2018). Thus, a variable that is currently attracting attention from researchers is the so-called “connectivity with nature” or “connection with nature,” defined as “the way in which a person includes the natural environment, nature, within the cognitive representations of oneself” (Schultz, 2002, p. 67) and implies the emotional connection that a person has or feels toward nature (Mayer and Frantz, 2004; Olivos and Clayton, 2017). Therefore, this is regarded as a construct that highlights the extent to which the natural environment is embedded within the self-image; that is, to what extent do people conceive of themselves as being linked to nature and build their sense of self by perceiving a feeling of similarity and belonging to the natural world. The formulation of this construct is due to the fact that, according to the extent to which the construction of the self includes the natural environment, this will determine both the type of environmental concerns that will prevail in the person, and the type of situations that will trigger the expression of specific pro-environmental behaviors (Mena and Olivos, 2018; Olivos-Jara and Aragonés, 2014). Schultz (2002) states that this inclusion hinges on an affective-emotional component that describes feelings of intimacy, closeness, and affection toward nature. This affectivity, in conjunction with the cognitive component, that is, the feeling of connection with nature, can be linked to the commitment to protect nature, acting in accordance with the interests of care, respect, and proper use of natural resources. Thus, it could be supposed that when a person decides to cast a vote in favor of the pro-environmental parties, the feeling of connection with nature would be present, which is a possibility that has not yet been explored in the literature.

### Statement of the Problem

In light of the literature reviewed, there is sufficient empirical evidence to suggest the existence of positive correlations between pro-environmental behavior, connectivity with nature, biospheric values, and environmental beliefs, as well as negative correlations among these variables and egocentric values (Olivos et al., 2014; Corral-Verdugo et al., 2016). However, these relationships have not been examined when the behavior under study is the pro-environmental vote.

It is clear that governments play an important role in protecting the environment. Given that these governments are elected by civil society, we believe that it is necessary to promote pro-environmental voting behavior, particularly if we consider that only a minority (of voters) believe that the environmental protection commitments adopted by political parties are very important when voting (Carreón et al., 2014). From this perspective, and given that there are barely any studies that analyze the key factors that, from a psychosocial point of view, should be considered when promoting pro-environmental voting behavior in society, we believe it is necessary to identify these factors. Thus, the first objective of the present research is to identify the psychosocial profile of the pro-environmental voter with respect to the variables that, according to the literature reviewed, could play a relevant role in this behavior, which are values, environmental beliefs, and connectivity with nature. As we have already indicated above, these variables are important for explaining environmental behaviors.

To establish this profile, we compared people who, in the last Spanish government elections, voted for a political party because it included environmental protection measures in its electoral program (pro-environmental voters) with those who voted for a political party without consideration of such issues (i.e., Non-pro-environmental voters). In Spain, the general elections are those in which citizens choose the members of the Congress of Deputies and the Senate, organizations that represent the Spanish people. These are held every 4 years, with the most recent taking place on April 28, 2019.

Based on the literature reviewed above, we expected to find support for the following hypotheses:

H.1: Participants who, during the last election, voted for a political party because its program included environmental protection measures, will show:
H.1.1: Greater adherence to biospheric values in comparison with other voters who did not take these measures into account.
H.1.2: Greater adherence to socio-altruistic values in comparison with those voters who did not take these measures into account.
H.1.3: Low adherence to egocentric values in comparison with those voters who did not take these measures into account.
H.1.4: Greater adherence to ecocentric beliefs than those voters who did not take these measures into account.
H.1.5: Low adherence to anthropocentric beliefs than those voters who did not take these measures into account.
H.1.6: A greater feeling of connection with nature than those voters who did not take these measures into account.

In addition, and given the lack of studies that analyze the determinants of voting behavior, a second objective of this study was to explore the predictive capacity of psychosocial variables that significantly characterize the profile of people who vote for parties with environmental proposals, particularly the behavior of those who cast a vote in favor of said political parties.

## Materials and Methods

### Participants

This study used an incidental sample that was initially composed of 550 participants, although 136 were excluded from the analyses because they either failed to respond to all of the items or because they were under 18 years of age.

Therefore, the final sample was made up of 414 Spanish participants with an age range between 18 and 67 years (mean age = 26.92, *SD* = 10.53). The participants were divided into two groups: (1) Pro-environmental voters, that is, those who during the last elections based their voting decision on whether the political party included pro-environment measures in its electoral program (*n* = 190, accounting for 45.9% of the total sample, with a gender distribution of 50.4% women and 38.6% men), and (2) Non-pro-environmental voters, that is, those other people who voted for a political party without considering whether pro-environment measures were included in its electoral program (*n* = 224, accounting for 54.1% of the sample and of which 49.6% were women and 61.4% were men) (Supplementary Data Sheet S1).

### Variables and Measurement Instruments

#### Values

Schwartz (1992) defines values as desirable, *trans*-situational goals that vary in importance and serve as guiding principles in people’s lives. Values here were measured using a total of 13 items, 11 of which were taken from the stock inventory of Schwartz (1992, 2009) and the remaining 2 from those proposed by Stern et al. (1999). The 13 values are classified into three dimensions: (a) Biospheric Values, understood as those values that imply a concern for non-human species and for the biosphere as a whole. It consists of five items, three taken from Schwartz’s inventory of values (-SVS-, 2009), (“union with nature,” “a world of beauty,” and “protecting the environment”) and the remaining two (“prevent pollution” and “respect for the land”) incorporated by Stern et al. (1999); (b) Socio-Altruistic values, defined as those values that imply concern for other people. It is composed of four items taken from the inventory of Schwartz values (-SVS-, 2009). These values are: “a world of peace,” “equality,” “social justice,” and “helping others”; (c) Egocentric values, referred to as those values that imply concern for oneself. These have also been extracted from Schwartz’s (SVS) value inventory -SVS- (2009) and in this case four values were considered: “authority,” “social power,” “healthy,” and “influential.” It should be clarified that the alpha coefficient presented for egocentric values does not include the “healthy” item, since eliminating this from the analysis increased the internal consistency of the dimension. We obtained an adequate internal consistency coefficient for the values (see Table 1).

**TABLE 1:** Means, standard deviations, and *t*-test results for pro-environmental voters and non-pro-environmental voters.

	*N* = 414	Pro-environmental voters (*n* = 190)	Non-pro-environmental voters (*n* = 224)				

	Alpha	Mean (*SD*)	Mean (*SD*)	*t*(df)	*p*	Cohen’s *d*	*r* (effect size)
**Biospheric values**	0.89	6.14 (0.99)	5.36 (1.45)	*t*_(__412__)_ = −6.23	0.000	0.62	0.29
**Egoistic values**	0.66	1.74 (1.60)	2.20 (1.58)	*t*_(__412__)_ = 2.98	0.003	–0.28	–0.14
**Socio-altruistic values**	0.74	6.40 (0.81)	5.88 (1.13)	*t*_(__412__)_ = −5.18	0.000	0.52	0.25
**Anthropocentrism**	0.73	5.08 (0.96)	4.63 (0.93)	*t*_(__412__)_ = −4.89	0.000	0.47	0.23
**Ecocentrism**	0.69	5.84 (0.73)	5.42 (0.84)	*t*_(__412__)_ = −5.32	0.000	0.53	0.25
**Connectivity with nature**	0.80	3.57 (0.56)	3.30 (0.60)	*t*_(__412__)_ = −4.56	0.000	0.46	0.22
**Environmental concern**	–	2.30 (0.93)	1.57 (1.28)	*t*_(__412__)_ = −6.53	0.000	0.65	0.31

**The coefficient of internal consistency (α) found for each variable is shown for the entire sample*.*

Participants were asked to evaluate to what extent each value was important as a guiding principle for their life, using a Likert-type response format with 9 points ranging from −1 (which indicates that the principle “opposes their values”) to +7 (which indicated that the value was considered to be “of supreme importance”). Operationally, each of the three dimensions is obtained from the average scores of the participants for each cluster of values. We thus obtained three scores, one for each value orientation.

#### Environmental Beliefs

Stern et al. (1999) and Stern (2000), define this variable as those general visions about the world, reflected in the beliefs that people express about their relationship with the environment and nature. To measure environmental belief systems, taken as the degree of awareness or concern for the environment, we used the revised version of the scale of the new environmental paradigm (NEP) proposed by Dunlap et al. (2000), adapted to the Spanish context by Vozmediano and San Juan (2005). This version consists of 11 items on a Likert-type response format to indicate the degree to which the person identifies with each statement, ranging from 1 (“strongly disagree”) to 7 (“strongly agree”). With this scale, it is possible to evaluate the two independent dimensions of ecocentrism and anthropocentrism. Anthropocentric beliefs are defined as those beliefs related to thinking that the human being is the owner of nature, and as such they can be shielded from its laws, valuing nature only on the basis of the benefits/costs that it implies for oneself. Ecocentric beliefs correspond to those beliefs that contemplate the existence of an imbalance in nature due to the actions of humans, and the need to respect the biosphere. The alpha coefficient obtained for each dimension is shown in Table 1.

#### Connectivity With Nature

Connectivity with nature was evaluated using the scale proposed by Mayer and Frantz (2004), adapted to the Spanish context by Olivos et al. (2011). It consists of 14 items, which measure the degree to which people feel, in general, part of the natural world. For this scale we obtained a high coefficient of internal consistency (see Table 1). The response to each item is given using a 5-point Likert-type response scale, ranging from +1 (strongly disagree) to +5 (strongly agree).

#### Pro-environmental Voters vs. Non-pro-environmental Voters

To evaluate this variable, an item was first used regarding whether they had exercised their right to vote in the last general elections held in Spain, with a dichotomous response format (yes/no). To assess whether the participant had exercised their right to vote by considering whether the party for which they voted included environmental protection measures in its electoral program (Pro-environmental Vote), this dichotomous response format (yes/no) was also used. More specifically, they had to answer the following question: “Have you voted in the last general election for a political party because it included measures for the protection of the environment in its electoral program?”. To strengthen the internal validity of this variable, we evaluated the degree to which the environment is of importance (concern) to the voters. This variable was evaluated using a single item: “I am worried about the environment,” with a Likert-type response scale (extracted from the scale of Gärling et al., 2003).

### Procedure

For collection of the data, a self-administered online questionnaire was used, consisting of the previously described scales along with questions related to sociodemographic variables (gender and age).

The participants were invited to participate voluntarily in the study. The call for participation was made through information posters that were placed in different areas of the city of Granada (Spain) and disseminated through various social networks. This poster mentioned the objective of the study and indicated that a requirement for participation was “to have voted in the last elections.” The information presented on the poster was as follows:

“We invite you to participate in an important study of the University of Granada, which aims to assess how certain psychological factors influence the voting decision. This consists of completing a questionnaire through an online platform, which you can access through the following link or by scanning the QR code. The study will take about 10 min to complete. Your participation will be voluntary and anonymous. The data we obtain from the study will be for the exclusive use of the research and will be treated confidentially.”

### Analysis

For analysis of the data, the statistical package SPSS vs. 24 was used, which allowed us to obtain correlations for the entire sample, and to analyze all of the variables — both independent and dependent — as well as to provide descriptive statistics and a comparison of means for value dimensions, anthropocentric beliefs, ecocentric beliefs and connection with nature between the two groups. For the comparison of means we used the Student’s *t*-test for independent samples. To address the first objective, i.e., to identify the psychosocial profile of the person who votes for a political party by considering whether its electoral program includes measures to protect the environment (“pro-environmental voters”), we considered the relevant variables (from the study of psychology) for explaining pro-environmental behavior. These variables are those related to environmental values and beliefs, as well as connectivity with nature. Two groups of voters were established: “Pro-environmental voters” and “Non-pro-environmental voters.” In addition, to address the second objective, a binomial regression analysis was carried out, since the dependent variable was of a dichotomous nature, taking as independent variables the three value dimensions (biospheric, socio-altruistic, and egoistic), environmental beliefs (ecocentric and anthropocentric), and the feeling of connectivity with nature. In this regression analysis, the effect of gender and age was controlled, taking them as covariates. In order to strengthen the internal validity of the dependent variable, a Student’s *t*-test was conducted to compare the differences in the means between the two groups of voters with regard to the degree of environmental concern.

## Results

The correlations between all the variables of the study, for the whole sample, can be observed in Table 2. The results revealed that in general the variable of pro-environmental vote is significantly related to the rest of the variables considered, and correlates positively with all variables except egoist values, with which the relationship is negative. It should be noted that there was a significant positive correlation between ecocentrism and anthropocentrism. Analysis of the difference in the means between the groups of voters (see Table 1) in terms of environmental concern revealed a significant difference between the participants who vote based on consideration for the environment and those who do not. The results obtained in relation to Hypotheses 1.1, 1.2, and 1.3 indicate that there are significant differences in terms of the three value orientations. In particular, the group of Pro-environmental voters showed a greater orientation toward biospheric and socio-altruistic values than Non-pro-environmental voters, as well as a lower tendency to adhere to egocentric values. The results therefore support these three hypotheses.

**TABLE 2:** Correlation coefficients found between all the variables considered.

	(1)	(2)	(3)	(4)	(5)	(6)	(7)	(8)
**(1) Biospheric values**	–	0.556**	−0.114*	0.419**	0.221**	0.519**	0.287**	0.464**
**(2) Socio-altruistic values**		–	−0.204**	0.326**	0.201**	0.298**	0.276**	0.397**
**(3) Egoistic values**			–	−0.025	−0.235**	−0.074	−0.146**	−0.148**
**(4) Ecocentrism**				–	0.356**	0.270**	0.271**	0.426**
**(5) Anthropocentrism**					–	0.048	0.257**	0.209**
**(6) Connectivity with nature**						–	0.231**	0.476**
**(7) Pro-environmental vote**							–	0.314**
**(8) Environmental concern**								–

**The correlation is significant at the 0.01 level; *the correlation is significant at the 0.05 level. The correlations involving the dependent variable (pro-environmental vote) were calculated using the Spearman coefficient, whilst the remaining correlations were calculated using the Pearson coefficient.

In relation to Hypotheses 1.4 and 1.5, the results revealed that there were significant differences between both groups of voters regarding ecocentric and anthropocentric beliefs. The Pro-environmental voters showed a greater adherence to ecocentric and anthropocentric beliefs than the group of Non-pro-environmental voters (see Table 2). Therefore, these findings support Hypothesis 1.4, but not Hypothesis 1.5.

These results are not entirely consistent with previous findings in the literature regarding the relationship between anthropocentrism and pro-environmental behavior. More specifically, the review of the literature on NEP shows that ecocentrism is positively related to a greater tendency to act in favor of the environment, while anthropocentrism is negatively related to pro-environmental behavior. Therefore, it might have been reasonable to expect our Pro-environmental voters to have a high degree of adherence to ecocentric beliefs, and low adherence to anthropocentric beliefs.

Finally, the results indicate that the pro-environmental voters have a greater feeling of connection with nature than non-pro-environmental voters, with this difference reaching statistical significance (see Table 1). Therefore, these results also provide support for Hypothesis 1.6.

In sum, the results found in relation to this first objective confirm that, indeed, pro-environmental voters show higher scores on the variables, that according to the literature, are characteristic of people who have a greater awareness and concern for the environment, which, in turn, results in a higher rate of pro-environmental behavior. However, since these variables do not necessarily influence provisional voting behavior in the same way, in the second objective we aimed to confirm whether the psychosocial variables that characterize the profile of participants that adhere to principles in favor of the pro-environmental vote could indeed predict or explain such behavior. To this end, a binary logistic regression analysis was conducted, taking voting behavior as a dependent variable and the various value dimensions, ecocentrism, anthropocentrism, and the feeling of connection with nature as independent variables. This analysis was carried out with the objective of exploring which variables act as determinants of pro-environmental voting behavior, in order to confirm their predictive validity.

In this regard, it should be noted that the index found for the omnibus test was less than 0.05, which indicates that the model helps to explain pro-environmental voting behavior, that is, that the independent variables taken into account explain the dependent variable. More specifically, the proportion of the dependent variable explained by this model ranged between 0.15 (Cox R2 and Snell) and 0.20 (Nagelkerke R2). Therefore, the model explained 20% of the dependent variable considered (Pro-environmental Vote). To analyze the goodness of the fit of the model, we must first pay attention to the Hosmer and Lemeshow Test, where a chi-Square value of 8.21 was found (*p* = 0.412), which translates into a first sample of goodness of fit of the model. However, following Rojo (2007), the coefficients of goodness of fit are not entirely reliable and therefore the classification table is the criterion that we normally must follow to indicate the goodness of fit of the model.

With the data of our sample, it appears that 72.6% of participants who did not vote for pro-environmental parties in the last elections were correctly classified by the model, while 27.4% were incorrectly classified. Likewise, 63.3% of the participants who voted by parties that include pro-environmental proposals in the last elections were correctly classified by the model, while 36.7% were not classified correctly. In total, with this model, 68.3% of the participants who voted (or not) for pro-environmental parties were correctly classified.

The most influential variables for predicting pro-environmental voting behavior (see Table 3) were ecocentric beliefs (*p* = 0.030) and anthropocentric beliefs (*p* = 0.014). These variables were significant at the 5% level. It should be noted that biospheric values (*p* = 0.056) were marginally significant. Of all of the variables, the one that showed the greatest strength for explaining pro-environmental party voting was that of ecocentric beliefs [its exponential of b -Exp (b) – is the one that moved furthest away from 1]. The variables that did not predict the pro-environmental vote were connectivity with nature (*p* = 0.078), egocentric values (*p* = 0.124), and socio-altruistic values (*p* = 0.102).

**TABLE 3:** Coefficients of the variables evaluated in the logistic regression.

	*B*	E.T.	Wald	df	Sig.	Exp(*B*)
Anthropocentrism	0.313	0.127	6.026	1	0.014	1.367
Ecocentrism	0.348	0.160	4.703	1	0.030	1.416
Biospheric values	0.235	0.123	3.649	1	0.056	1.264
Socio-altruistic values	0.237	0.144	2.680	1	0.102	1.267
Connectivity with nature	0.373	0.212	3.105	1	0.078	1.453
Egoistic values	–0.111	0.072	2.369	1	0.124	0.895
Constant	–7.626	1.297	34.574	1	0.000	0.000

## Discussion

In this paper we understand pro-environmental voting behavior to be an indirect environmental protection action that, whilst being within the domain of socio-political behaviors, could also fall within the category of environmental behavior. Given that relatively few empirical studies have analyzed the main determinants of pro-environmental voting behavior from a psychosocial point of view, our aim was to explore if there were differences between the so-called pro-environmental voters and non-pro-environmental voters in terms of cognitive and emotional variables that are closely linked to environmental behavior. This was the first objective of the present work. More specifically, we explored the differences between both groups in terms of their adherence to biospheric, socio-altruistic, and egocentric values, as well as in terms of their environmental belief system (ecocentrism vs. anthropocentrism) and the extent to which they feel a connection with nature. According to our results, in comparison with non-pro-environmental voters, those people who during the most recent elections, made their decision to vote for a political party because the latter included pro-environmental measures in their electoral program (pro-environmental voters), have a greater predisposition toward biospheric and altruistic social values, as well as a greater connection with nature whilst they adhere to a lesser extent to egocentric values. These results are in line with those obtained in previous studies examining other environmental behaviors (e.g., Aguilar-Luzón et al., 2006, 2008; Amérigo and González, 2008; Olivos-Jara and Aragonés, 2014; López et al., 2015). These findings appear to confirm that when comparing the two types of voters (pro-environmental voters vs. non-pro-environmental voters) studied here, there are differences in the variables related to the environmental behavior evaluated. In this regard, and in relation to the factors that influence pro-environmental voting behavior, our results are consistent with others found in the literature, confirming the important role of values when studying voting behavior (Caprara et al., 2006, 2008; Schwartz et al., 2010), and in our case, those values of a biospheric nature are particularly important. These results confirm the ideas of some authors in relation to the so-called “ecological citizenship” (Dobson, 2005), in which these behaviors are not based on motives associated with egocentric or selfish values but must instead be subject to altruistic and biospheric value orientations.

Moreover, we have found that pro-environmental voters have a greater connectivity with nature in comparison with non-pro-environmental voters. In this regard, as we expected, the people with the greatest connectivity are those who chose to cast their vote in favor of parties that include environmental protection measures among the proposals of their electoral program, which is consistent with the findings of previous studies that have analyzed the association between connectivity with nature and other pro-environmental behaviors (Amérigo and García, 2014). We believe that these results contribute toward explaining how a feeling of connection with nature is related to a feeling of responsibility and care for other creatures and the natural environment, which is manifest through the expression of values that seek the preservation of nature (biospheric) and the good of others (altruists). However, connectivity with nature, despite being more present in pro-environmental voters than in non-pro-environmental voters, did not turn out to be a strong predictor of voting behavior. As far as predictive capacity is concerned, our findings do not appear to be in line with the suggestions offered by other authors regarding pro-environmental behavior. In particular, it has been stated that people who feel the strongest connections with nature express greater concern for the biosphere and are more likely to engage in behaviors that help to protect the environment (Schultz et al., 2004; Olivos-Jara and Aragonés, 2014). However, our findings are in accord with those reported by Gkargkavouzi et al. (2019), who conclude that in the case of policy support and transportation choices, environmental concerns explained more variance than the other constructs. However, connectedness to nature and ecological worldview were more predictive than environmental concerns in other domains of behavior, i.e., civic actions, recycling, household behaviors, and consumerism.

Further, when the predictive capacity of the variables was analyzed with respect to the pro-environmental voting decision, the results revealed that high scores on ecocentric and anthropocentric beliefs predict behavior in favor of the pro-environmental vote. These results are not consistent with those of Vozmediano and San Juan (2005), since these authors found that ecocentrism is positively related to pro-environmental behavior, while anthropocentrism is negatively related to such behavior. However, our results are in line with those reported by Dunlap et al. (2000), who assume that both beliefs (anthropocentric and ecocentric) constitute a single dimension, and can thus show positive relationships with environmental behaviors.

We believe the fact that anthropocentric beliefs not only characterize the profile of the pro-environmental voter, but also contribute toward predicting the decision to vote pro-environmental could be taken to reflect a shift in the way in which society views the relationship between people and the environment, constituting evidence in favor of the postulates of the New Paradigm of Human Interdependence (Corral-Verdugo et al., 2008). Our results point to the existence of a high correlation between ecocentrism and anthropocentrism, and thus support the approach adopted by this new paradigm.

This paradigm postulates a belief system in favor of the environment that integrates both ecocentric and anthropocentric beliefs within a single dimension. In this way, the belief that the environment needs man for its preservation (ecocentrism), coexists with the notion that the human being requires nature in order to survive (anthropocentrism) (Corral-Verdugo, 2010; Hernández et al., 2012).

This approach, derived from the New Paradigm of Human Interdependence — that is, the notion that it is possible to satisfy human needs without renouncing care of the environment and without austerity measures — is consistent with the foundations of sustainable development (Corral-Verdugo and García, 2014). Thus, some authors such as Hernández and Suárez (2006) point out that in order for proenvironmental behavior to be maintained over time, the work of both researchers and professionals should consider human well-being.

Some authors have linked this paradigm of thought with the achievement of actions that entail the protection of the environment (Amérigo and García, 2014; Corral-Verdugo and García, 2014). Thus, the results found here appear to be in line with this integrated conception of environmental beliefs. Consequently, this raises the question of whether the dichotomy traditionally held by the literature on environmental beliefs continues to be perceived in today’s society (Dunlap et al., 2000).

From this perspective, the relevance of anthropocentric beliefs (along with ecocentric beliefs) in predicting the behavior of the pro-environmental voter is evident, since both types of beliefs can support pro-environmental behavior. Therefore, in light of our results, the behavior of casting a pro-environmental vote can be favored by a high level of adherence to both ecocentrism and anthropocentrism (Kaida and Kaida, 2016).

Whilst our findings can contribute to improving knowledge about the psychosocial factors that characterize pro-environmental voters, and can therefore help to predict pro-environmental voting behavior, before drawing firm conclusions on this issue we should point out some of the possible limitations of this study. In particular, it is important to emphasize that the social desirability of the participants has not been controlled. We must bear in mind that behavior against the environment or passivity in the face of environmental degradation is something that might be denied by the majority of the population. Although the presence of this bias is always assumed when working in this field, it would be worthwhile to attempt to avoid this in future research (Amérigo and Aragonés, 2010). A further possible limitation of this study is the age of the participants. According to the latest Eurobarometer (2019), in comparison with older voters, younger voters are more likely to say that combating climate change and protecting the environment was an issue that informed their voting decision (45% of those aged under 25 compared with 34% of those aged 55 or over), so these results should be interpreted with some caution.

Moreover, the priority that the participants assign to the preservation of the environment has not been taken into account, since they have only been asked if they consider this issue when casting their vote. This issue is worth noting since the current situation in Spain is one in which there is a high demand for employment and a low supply of jobs, constant cuts in social benefits, and cases of corruption that impoverish the state. Thus, it is highly probable that the population would have cast their vote in the last general election on the basis of considerations of a material nature such as employment and security as opposed to post-material considerations such as the environment, equality, and others (Calvo, 2017). We think that this aspect should be considered in future investigations since it could provide more specific information regarding the differences between the voters.

In summary, and in conclusion, the results of this study suggest that biospheric value orientations and anthropocentric and ecocentric beliefs contribute to the prediction of pro-environmental voting behavior. However, the percentage of variance explained (20%) by these variables is low, which suggests the need for further research to identify other variables of interest for the prediction of this behavior.

We believe that our results could help to explain why there are people who, with their voting behavior, aim to support a political system that contemplates the restoration and care of environments, connecting with their communities through the care of the Earth (Maller et al., 2008). However, we also believe that it is necessary to conduct a wider range of investigations that could either confirm or refute our results, as well as to expand the set of possible factors that may be relevant in the expression of pro-environmental voting behavior.

Voting behavior in general has traditionally been studied from sociopolitical perspectives. Given that in our study we consider that the behavior of the pro-environmental voter particularly implies an indirect environmental action (casting a vote in favor of political parties that include environmental protection measures in their proposals for governing a country), it is of fundamental importance to adopt a study approach that is derived from psychosocial models. However, this does not mean that pro-environmental voting behavior should not be addressed from sociopolitical models, and this is indeed an issue that could be addressed in future research developments.

We believe that environmental issues have taken on a very important role in the current panorama in all its aspects: political, social, and economic, and perhaps in the future this scenario is one that could lead to a better balance between the needs of humans and the care of the environment.

In short, this paper has described factors such as beliefs (anthropocentric and ecocentric) and biospheric values that could explain the behavior of the pro-environmental voter. More specifically, our results show that among all the variables considered, those that best predict the pro-environmental voting behavior are beliefs. Further, it is also important to note the potential applied relevance of our findings. Prior to launching political campaigns, thanks to this type of research, political parties and their candidates could have a greater opportunity to become more familiar with the citizens they serve and to direct their electoral programs toward achieving greater collective environmental awareness. In this regard, we believe that our results could be used to implement smart strategies aimed at winning the votes of the so-called pro-environmental citizenship. These could include, for example, political strategies that promote biospheric values or generate greater environmental awareness and beliefs with the ultimate goal of ensuring that today’s children — who will be the adults of tomorrow — are respectful of the environment in which they live.

## Data Availability Statement

All datasets generated for this study are included in the article/Supplementary Material.

## Ethics Statement

The studies involving human participants were reviewed and approved by Research Ethics Committee of the University of Granada (Spain) with the file number on 194/CEIH/2016. The ethics committee waived the requirement of written informed consent for participation.

## Author Contributions

MA-L: substantial contributions to the conception or design of the work, analysis and interpretation of data for the work, drafting the work, and providing approval for publication of the content. BC: substantial contributions to the acquisition, analysis or interpretation of data for the work, drafting the work, and providing approval for publication of the content. AC-S: substantial contributions to the acquisition, analysis or interpretation of data for the work, and providing approval for publication of the content. PC: substantial contributions to the analysis of data for the work and providing approval for publication of the content.

## Conflict of Interest

The authors declare that the research was conducted in the absence of any commercial or financial relationships that could be construed as a potential conflict of interest.
